# Prevalence, awareness, treatment, and control of hypertension in Northern China: a cross-sectional study

**DOI:** 10.1186/s12872-021-02333-7

**Published:** 2021-11-04

**Authors:** Xiaoqian Xu, Han Bao, Zixuan Tian, Hao Zhu, Lige Zhu, Liwei Niu, Tao Yan, Hairong Dong, Xin Fang, Xingguang Zhang

**Affiliations:** 1 School of Public Health, Inner Mongolia Medical University, Hohhot, Inner Mongolia People’s Republic of China; 2 Department of Nephrology, Inner Mongolia People’s Hospital, Hohhot, Inner Mongolia People’s Republic of China; 3 Department of Clinical Laboratory, Hohhot First Hospital, Hohhot, Inner Mongolia People’s Republic of China

**Keywords:** Hypertension epidemiology, Blood pressure, China, Prevalence, Control, Associated factors

## Abstract

**Background:**

Hypertension has always been a worldwide health concern. The purpose of this study was to investigate the prevalence, awareness, treatment, and control rates of hypertension among adult residents of northern China, where people usually have a high-fat, high-salt diet and heavy alcohol consumption.

**Methods:**

Through the Early Screening and Comprehensive Intervention Project for High Risk Groups of Cardiovascular Diseases in the Inner Mongolia of northern China, we collected data of 70,380 residents, from September 2015 to June 2017. We assessed the prevalence, awareness, treatment, and control of hypertension in the total population and sub-populations. Multivariable logistic regression analyses were used to identify the factors associated with the prevalence and control of hypertension.

**Results:**

Among participants, only 13.4% had optimal blood pressure levels. About 55.7% (95% confidence interval (CI) = 55.3–56.1%) of the participants had hypertension. In addition, the awareness, treatment, control and control under-treatment rate of hypertension were 52.8% (95%CI = 52.3–53.3%), 43.3% (95%CI = 42.8–43.8%), 8.6% (95%CI = 8.3–8.9%) and 19.8% (95%CI = 19.2–20.4%), respectively. Multivariable logistic regression showed that older, male, Han, living in rural areas, current drinker, not married, lower educational level, lower annual income, diabetes, obesity, and dyslipidemia were more likely to be suffered from hypertension (*P* < 0.05). Controlled hypertension was less common in those younger, Mongol, not married, farmer, current drinker, lower educational level, obesity, diabetes, without prior CHD, and without prior CVD (*P* < 0.05).

**Conclusion:**

Among populations aged 35–75 years in Northern China, more than half have hypertension, fewer than one-tenth have successfully controlled hypertension, and fewer than one-fifth of hypertension patients receiving treatment have controlled hypertension.

## Background

Hypertension is a leading health risk [1, 2]. Hypertension contributes to 51% of stroke deaths and 45% of ischemic heart disease deaths worldwide [3] and causes more than 7 million premature deaths each year [4]. The Global Burden of Disease Study 2019 indicated that high systolic blood pressure (SBP) accounted for 10.8 million deaths and was responsible for the largest number of all-cause deaths [5]. Studies have shown that the global prevalence of hypertension has risen continuously in recent decades, especially in low-income and middle-income countries [2]. It is estimated that 26.4% (972 million) of adults had hypertension in 2000, and the number in 2025 is predicted to increase by about 60%, to a total of 1.56 billion globally [3]. The number of Chinese adult patients with hypertension increased from 153 million in 2000 to more than 270 million in 2012 [4]. A national study during 2014–2017 showed that 44.7% of Chinese adults aged 35–75 years had hypertension [6]. However, the level of hypertension management remains suboptimal in China, with fewer than half of patients with hypertension in China receiving treatment and fewer than one-fifth with controlled [7, 8].

Studies have shown that the prevalence of hypertension in southern China is lower than that in the northern part of the country [7]. Because Inner Mongolia lies across a large part of northern China, it is somewhat representative of this region, where people have a high-fat, high-salt diet and heavy alcohol consumption [9]. However, previous studies have covered a limited area and have included small sample size, and most studies have not linked hypertension with demographic characteristics and clinical variables of sub-populations [10–13]. The disease burden of hypertension in northern China remains unclear and identifying adverse factors involved in hypertension is urgent. Thus, obtaining reliable information about the prevalence, awareness, treatment, and control of hypertension is critical for further prevention and control of this disease and diseases secondary to hypertension.

Accordingly, in this study, we aimed to provide updated and reliable data on the hypertension prevalence, awareness, treatment, and control of the total population and sub-populations in northern China’s Inner Mongolia, and to identify the factors associated with the prevalence and control of hypertension.

## Materials and methods

### Study population

The study comes from the Early Screening and Comprehensive Intervention Project for High-Risk Groups of Cardiovascular Diseases in Inner Mongolia. Multistage cluster sampling method was used to recruit the study population, from September 2015 to June 2017. In the first stage, we selected 6 sites (Hohhot, Wuhai, Chifeng, Erdos, Hulun Buir, and Xingan League) from Inner Mongolia, according to the economic level, geographical location, and distribution of ethnic minority nationalities, etc. In the second stage, the local Centre for Disease Control and Prevention then identified one district or county at each site based on the urban–rural distribution and population stability. In the third stage, we selected two or three urban residential communities or rural villages from each of the districts or counties according to the size of the communities or villages. In the final stage, invite the residents from each of the communities or villages to participate in the study through extensive publicity on television, broadcast and in the newspaper. The study population comprised 70,380 participants who were aged 35–75 years and lived there for at least 6 of the previous 12 months. This project was approved by the ethics committee of Fuwai Hospital Chinese Academy of Medical Sciences (approval number: 2014-574, approval date: July 2014), and all participants gave their written informed consent.

### Measurement

We used a self-designed questionnaire and trained medical personnel conducted face-to-face interviews. The questionnaire mainly included demographic characteristics such as age, ethnic group, education, marital status, annual Income, health insurance, history of the disease, and lifestyle factors including smoking and drinking. Information on height, weight, blood pressure, fasting blood lipids, and fasting blood glucose was obtained in physical or laboratory examinations.

For each participant, we measured blood pressure by electronic blood pressure monitor (Omron HEM-7430; Ormon Corporation, Kyoto, Japan) two times on the right upper arm after at least 5 min of rest in a seated position. If the SBP or DBP difference is > 5 mmHg, the third time is measured, and the average blood pressure value of the two or three readings was used. We measured the height and weight of participants by calibrated height and weight instrument. Before the measurement, the subject removed the shoes and hats, took out the weight in the pocket, stood upright when standing, straightened the back, and naturally sagged. The eyes are looking straight ahead and the values are recorded after the values are stable. After at least 10 h of overnight fasting, venous blood samples were collected for the measurement of blood glucose and blood lipids. Blood glucose was measured by a glucose analyzer (BeneCheck PD-G001-2, Taiwan, China). A blood lipid test that measured triglyceride (TG), total cholesterol (TC), high-density lipoprotein cholesterol (HDL-C), and low-density lipoprotein cholesterol (LDL-C) was performed by a rapid lipid analyzer (CardioChek PA Analyzer; Polymer Technology Systems, Indianapolis, Indiana, USA).

### Definition

Hypertension referred to an average SBP ≥ 140 mmHg and/or average diastolic blood pressure (DBP) ≥ 90 mmHg, or self-reported use of antihypertensive medications in the past 2 weeks. Awareness referred to a self-reported hypertension history or self-reported use of antihypertensive drugs among patients with hypertension. Treatment of hypertension referred to the use of antihypertensive medication among participants with hypertension. Control of hypertension referred to an average SBP < 140 mmHg and an average DBP < 90 mmHg among patients with hypertension. Control under treatment referred to an average SBP < 140 mmHg and average DBP < 90 mmHg after using antihypertensive medication among patients with hypertension. According to the 2018 Revision of Chinese Guidelines for Hypertension Prevention and Control [14], optimal blood pressure is defined as an average SBP 90–119 mmHg and DBP 60–79 mmHg. High–normal, stage 1, stage 2, and stage 3 are defined as average SBP 120–139 mmHg and/or DBP 80–89 mmHg, SBP 140–159 mmHg and/or DBP 90–99 mmHg, SBP 160–179 mmHg and/or DBP 100–109 mmHg, and SBP ≥ 180 mmHg and/or DBP ≥ 110 mmHg, respectively. If SBP and DBP had different grades, the higher grade was chosen.

Body mass index (BMI) referred to weight in kilograms (kg) divided by height in meters squared (m^2^). Obesity [15] referred to a BMI of at least 28 kg/m^2^. Diabetes [16] referred to the use of hypoglycemic medication or a measured fasting blood glucose level ≥ 7.0 mmol/L or non-fasting blood glucose level ≥ 11.1 mmol/L. Dyslipidemia [17] referred to either TG ≥ 2.26 mmol/L, TC ≥ 6.22 mmol/L, LDL-C ≥ 4.14 mmol/L, HDL-C < 1.04 mmol/L, or self-reported use of lipid-lowering drugs. Current drinker referred to drinking alcohol at least 1 time/month in the previous 1 year. Current smoker referred to smoking at least 1 cigarette every day.

### Statistical analysis

Statistical analyses were performed using SAS version 9.4 (SAS Institute, Cary, NC, USA). Based on data of the sixth national census of China 2010 (National Bureau of Statistics), the age- and sex-standardized rates of hypertension prevalence at the national level were calculated by the direct method. Continuous variables were expressed as mean and standard deviations (SD) and compared using the analysis of *t-*test*, t*′ test, or Wilcoxon rank sum test. Categorical variables were reported as numbers and proportions. Proportions were compared using the Pearson Chi-square test or Trend Chi-square tests. To analyze the association between individual characteristics and prevalence as well as control of hypertension, multivariable logistic regression models was built using hypertension prevalence and control as dependent variable (No = 0, Yes = 1). Independent variables with *P* value ≤ 0.10 in the univariable analysis were then included in the multivariable logistic regression model, performing in stepwise manner in the final multivariable logistic regression model via the Forward: LR method. Estimating adjusted odds ratios (ORs) and 95% CI using multivariable logistic regression. The independent variables and their coding are shown in Table 1. A two-sided *P* value < 0.05 was considered statistically significant.

**Table 1:** Independent variables and their coding

Variable	Categories
Sex	Male = 1 (reference), female = 2
Location of residence	Rural = 1 (reference), urban = 2
Marital status	Not married = 1 (reference), married = 2
Farmer	No = 1 (reference), yes = 2
Health insurance	No = 1 (reference), yes = 2
Diabetes	No = 1 (reference), yes = 2
Obesity	No = 1 (reference), yes = 2
Dyslipidemia	No = 1 (reference), yes = 2
Current smoker	No = 1 (reference), yes = 2
Current Drinker	No = 1 (reference), yes = 2
Prior CVD	No = 1 (reference), yes = 2
Prior CHD	No = 1 (reference), yes = 2
Ethnicity	Han = 1 (reference), Mongol = 2, others = 3
Age (years)	35–44 = 1 (reference), 45–54 = 2, 55–64 = 3, 65–75 = 4
Annual household income (yuan)	Low (< 10,000) = 1 (reference), middle (10,000–50,000) = 2, high (> 50,000) = 3
Educational level	Low (primary school or lower) = 1 (reference), middle (middle school) = 2, high (high school) = 3

## Results

A total of 70,380 participants aged 35–75 years were included in the study. Among participants, 58.0% were female, the mean age were 54.4 (SD: 9.4) years. The mean SBP and DBP was 140.5 (SD: 20.9) mmHg and 84.8 (SD: 11.6) mmHg, respectively. The prevalence of diabetes was 19.5% and the mean BMI was 25.8 (SD: 3.6) kg/m^2^ (Table 2).

**Table 2:** Baseline characteristics of the study population

Characteristics	All subjects (*N* = 70,380)
Age (years), mean ± SD	54.4 ± 9.4
Blood pressure (mmHg), mean ± SD	
SBP	140.5 ± 20.9
DBP	84.8 ± 11.6
BMI (kg/m^2^), mean ± SD	25.8 ± 3.6
Sex, n (%)	
Male	29,539 (42.0)
Female	40,841 (58.0)
Location of residence, n (%)	
Rural	48,695 (69.2)
Urban	21,685 (30.8)
Marital status	
Married	64,203 (91.2)
Not married	6177 (8.8)
Ethnicity, n (%)	
Han	63,172 (89.8)
Mongolian	6086 (8.6)
Others	1122 (1.6)
Annual household income, n (%)	
< 10,000 (yuan)	19,716 (28.0)
10,000–50,000 (yuan)	36,980 (52.6)
> 50,000 (yuan)	8044 (11.4)
Unknown	5640 (8.0)
Educational level, n (%)	
Primary school or lower	28,466 (40.5)
Middle school	30,576 (43.4)
High school or above	7814 (11.1)
Unknown	3524 (5.0)
Farmer, n (%)	33,186 (47.2)
Health insurance, n (%)	61,139 (86.9)
Diabetes, n (%)	13,707 (19.5)
Dyslipidemia, n (%)	27,220 (38.7)
Obesity, n (%)	17,498 (24.9)
Current smoker, n (%)	17,624 (25.0)
Current drinker, n (%)	19,089 (27.1)

Data are reported as n (%) or mean ± SD. *SD* Standard deviation, *SBP* systolic blood pressure, *DBP* diastolic blood pressure, *BMI* body mass index

### Mean blood pressure levels and distribution of blood pressure

**Fig. 1:**
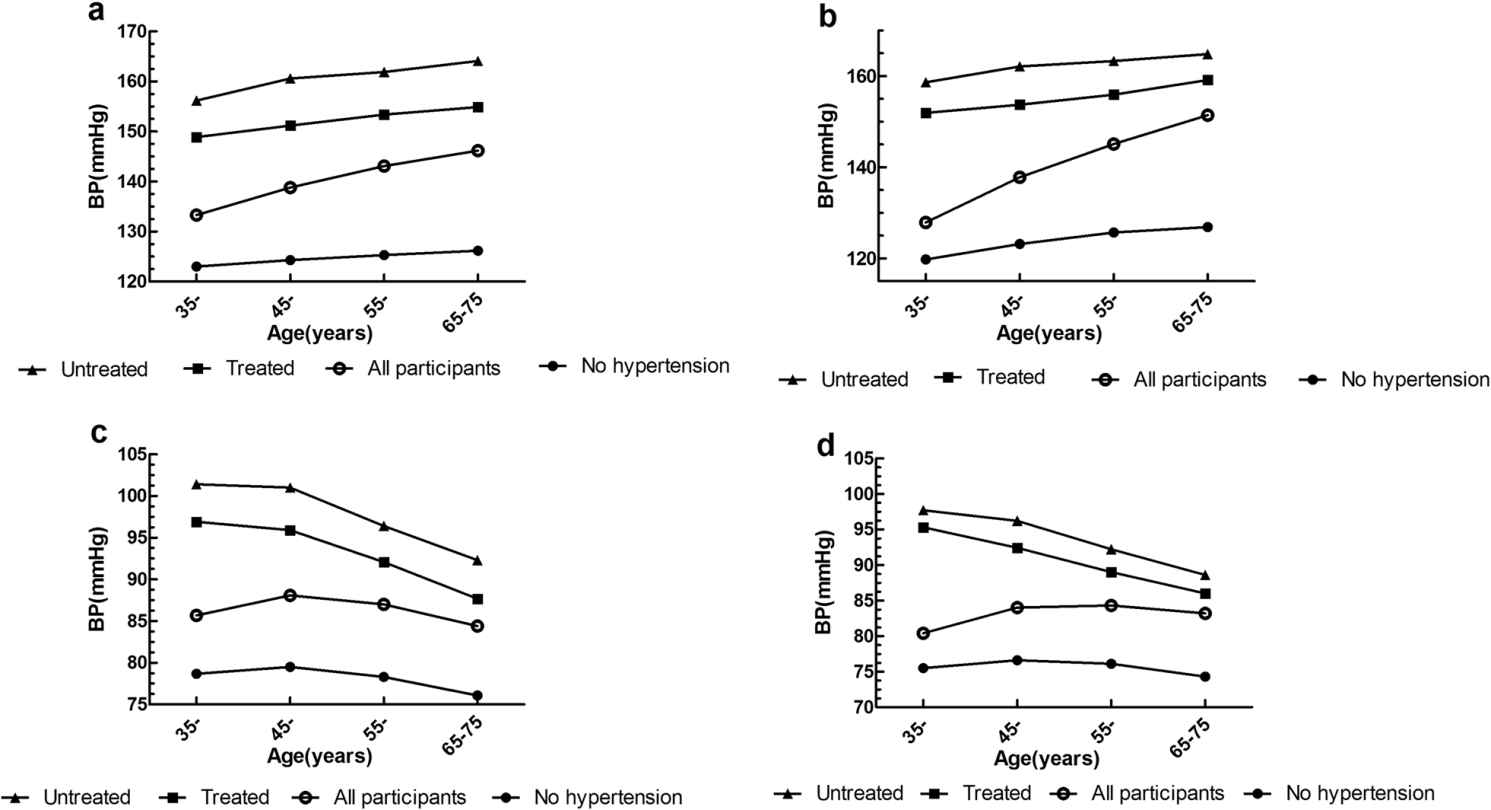
The linear trend of SBP and DBP with age in Northern China population of 35–75 years old, by sex. **a** SBP of male. **b** SBP of female. **c** DBP of male. **d** DBP of female. *SBP* Systolic blood pressure, *DBP* diastolic blood pressure

Figure 1 shows sex- and age-specific mean blood pressure values for all participants and for 3 subgroups defined by hypertension status. The mean blood pressure in our study was 140.5/84.4 mmHg (male 140.7/86.7 mmHg and female 140.4/83.4 mmHg). Overall, the mean SBP increased gradually with age throughout the entire age range, whereas the mean DBP decreased in the 65–75 age group. Overall, the mean SBP and DBP among participants with no hypertension were 31.0 (95%CI = 30.6–31.3) mmHg and 13.6 (95%CI = 13.4–13.8) mmHg lower than the corresponding values among those with treated hypertension, and 38.3 (95%CI = 37.8–38.9) mmHg and 18.3 (95%CI = 17.9–18.6) mmHg lower than the corresponding values for those with untreated hypertension. The overall SBP and DBP differences between treated and untreated participants with hypertension were 7.4 (95%CI = 6.8–8.0) mmHg (*t*′ =  − 20.644, *P* < 0.001) and 4.6 (95%CI = 4.2–5.0) mmHg (*t*′ =  − 22.999, *P* < 0.001), respectively.

**Fig. 2:**
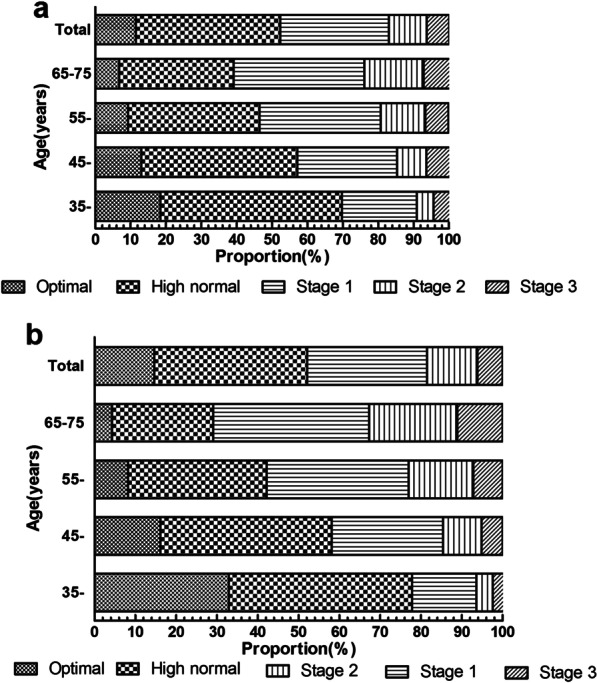
Percentage distribution of blood pressure levels in Northern China population of 35–75 years old, by sex and age. **a** Male. **b** Female

Figure 2 shows the percentage distribution of blood pressure levels among study participants. Overall, only 13.4% of the study population (11.5% of male and 14.7% of female) had optimal blood pressure, whereas nearly two-fifths (38.8%) had high normal blood pressure. The prevalence of stage 1, 2, and 3 hypertension was 30.7%, 10.7%, and 6.3% in male and 29.4%, 12.3%, and 6.2% in female, respectively. With increasing age, the percentage of optimal and high normal blood pressure decreased whereas the proportion of stage 1, stage 2, and stage 3 hypertension increased (Trend Chi-square tests: male, *x*^2^ = 999.904, *P* < 0.001; female, *x*^2^ = 41,666.177, *P* < 0.001).

### Prevalence, awareness, treatment, and control of hypertension

Table 3 shows the prevalence, awareness, treatment, control and control under treatment of hypertension by sub-populations. The crude rates of prevalence awareness, treatment, control and control under treatment for hypertension was 55.7% (95%CI = 55.3–56.1%), 52.8% (95%CI = 52.3–53.3%), 43.3% (95%CI = 42.8–43.8%), 8.6% (95%CI = 8.3–8.9%) and 19.8% (95%CI = 19.2–20.4%), respectively. The national standardized prevalence rate was 49.4% (95%CI = 49.0–49.8%). The population subgroups varied with respect to hypertension prevalence (range: 32.6–73.4%), awareness (range: 35.1–76.2%), treatment (range: 24.9–64.6%), control (range: 3.9–20.4%), and control under treatment (range: 11.2–31.6%).

**Table 3:** Prevalence, awareness, treatment, control, and control under treatment of hypertension among different groups, % (95%CI)

Groups	Prevalence	Awareness	Treatment	Control	Control under treatment
Crude rates	55.7 (55.3–56.1)	52.8 (52.3–53.3)	43.3 (42.8–43.8)	8.6 (8.3–8.9)	19.8 (19.2–20.4)
Sex					
Male	57.4 (56.8–58.0)	49.7 (48.9–50.4)	39.6 (38.9–40.3)	8.2 (7.8–8.6)	20.8 (19.8–21.7)
Female	54.5 (54.0–54.9)	55.2 (54.5–55.8)	46.1 (45.4–46.7)	8.8 (8.5–9.2)	19.2 (18.4–20.0)
Ethnicity					
Han	56.0 (55.6–56.4)	53.6 (53.1–54.1)	44.2 (43.7–44.7)	8.9 (8.6–9.2)	20.2 (19.6–20.8)
Mongol	52.7 (51.4–53.9)	44.6 (42.9–46.4)	34.2 (32.6–35.9)	5.1 (4.4–5.9)	15.0 (12.9–17.2)
Others	53.2 (50.3–56.1)	48.9 (44.9–52.9)	38.9 (35.0–42.8)	6.0 (4.1–7.9)	15.5 (10.9–20.2)
Age (years)					
35–44	32.6 (31.7–33.4)	35.1 (33.6–36.6)	24.9 (23.5–26.2)	5.3 (4.6–6.0)	21.4 (18.8–24.0)
45–54	50.6 (50.0–51.3)	47.9 (47.1–48.8)	38.1 (37.2–38.9)	8.0 (7.5–8.5)	21.0 (19.8–22.2)
55–64	64.2 (63.6–64.8)	56.9 (56.1–57.7)	47.8 (47.0–48.5)	9.5 (9.0–9.9)	19.8 (18.9–20.7)
65–75	73.4 (72.5–74.2)	60.9 (59.9–62.0)	51.6 (50.5–52.7)	9.3 (8.7–9.9)	18.0 (16.9–19.2)
Marital status					
Married	55.1 (54.7–55.5)	53.0 (52.5–53.5)	43.4 (42.9–44.0)	8.9 (8.6–9.2)	20.4 (19.8–21.0)
Not married	62.0 (60.8–63.2)	50.9 (49.3–52.5)	41.8 (40.3–43.4)	5.9 (5.2–6.7)	14.2 (12.5–15.9)
Farmer					
Yes	56.7 (56.2–57.3)	51.8 (51.1–52.5)	41.4 (40.7–42.1)	7.6 (7.2–8.0)	18.4 (17.5–19.3)
No	54.8 (54.2–55.3)	53.8 (53.1–54.4)	45.1 (44.4–45.7)	9.5 (9.1–9.9)	21.0 (20.2–21.8)
Location of residence					
Rural	56.0 (55.6–56.5)	52.5 (51.9–53.1)	42.4 (41.8–43.0)	8.3 (8.0–8.6)	19.5 (18.8–20.3)
Urban	54.9 (54.3–55.6)	53.6 (52.7–54.5)	45.3 (44.4–46.2)	9.2 (8.7–9.8)	20.4 (19.3–21.5)
Educational level					
Primary school or lower	58.8 (58.3–59.5)	54.3 (53.6–55.1)	44.3 (43.6–45.1)	8.0 (7.6–8.4)	18.1 (17.2–19.0)
Middle school	54.4 (53.8–54.9)	53.2 (52.4–53.9)	44.0 (43.2–44.7)	9.5 (9.0–9.9)	21.5 (20.6–22.4)
High school or above	47.8 (46.7–48.9)	49.8 (48.2–51.4)	40.1 (38.5–41.7)	9.5 (8.5–10.4)	23.6 (21.5–25.8)
Annual income (RMB)					
< 10,000	58.6 (58.0–59.3)	53.7 (52.8–54.6)	42.8 (41.9–43.7)	8.2 (7.7–8.7)	19.1 (18.0–20.2)
10,000–50,000	54.4 (53.9–54.9)	52.9 (52.2–53.6)	44.2 (43.5–44.9)	9.0 (8.6–9.4)	20.3 (19.5–21.1)
> 50,000	54.2 (53.1–55.3)	53.8 (52.3–55.3)	43.4 (41.9–44.9)	9.2 (8.4–10.1)	21.3 (19.4–23.1)
Health insurance					
Yes	55.5 (55.1–55.9)	53.4 (52.9–53.9)	43.8 (43.3–44.3)	8.8 (8.5–9.1)	20.0 (19.3–20.6)
No	56.9 (55.9–57.9)	49.0 (47.7–50.4)	39.9 (38.6–41.3)	7.4 (6.7–8.1)	18.6 (17.0–20.3)
Diabetes					
Yes	70.2 (69.4–70.9)	60.2 (59.2–61.2)	50.8 (49.8–51.8)	9.6 (9.1–10.2)	19.0 (17.9–20.1)
No	52.2 (51.8–52.6)	50.4 (49.8–51.0)	40.8 (40.3–41.4)	8.2 (7.9–8.5)	20.1 (19.4–20.9)
Obesity					
Yes	71.4 (70.7–72.0)	60.7 (59.8–61.5)	51.2 (50.3–52.1)	8.9 (8.4–9.4)	17.4 (16.5–18.4)
No	50.5 (50.1–50.9)	49.1 (48.5–49.7)	39.6 (39.0–40.2)	8.4 (8.1–8.7)	21.2 (20.5–22.0)
Dyslipidemia					
Yes	62.6 (62.0–63.2)	55.6 (54.8–56.3)	46.2 (45.5–47.0)	8.9 (8.4–9.3)	19.2 (18.2–20.2)
No	51.3 (50.8–51.8)	50.7 (50.0–51.3)	41.0 (40.4–41.7)	8.3 (8.0–8.7)	20.3 (19.4–21.3)
Current smoker					
Yes	53.2 (52.5–54.0)	50.7 (49.7–51.7)	40.8 (39.8–41.8)	9.4 (8.8–10.0)	23.0 (21.7–24.3)
No	56.6 (56.1–56.9)	53.5 (52.9–54.0)	44.1 (43.5–44.6)	8.3 (8.0–8.6)	18.9 (18.2–19.5)
Current drinker					
Yes	57.0 (56.3–57.7)	46.4 (45.5–47.4)	34.9 (34.0–35.8)	6.3 (5.9–6.8)	16.0 (14.8–17.1)
No	55.2 (54.8–55.6)	55.3 (54.7–55.8)	46.5 (45.9–47.1)	9.4 (9.1–9.8)	20.3 (19.6–21.0)
Prior CVD					
Yes	72.7 (71.2–74.2)	72.0 (70.2–73.7)	60.2 (58.2–62.1)	14.5 (13.1–15.9)	24.1 (21.9–26.3)
No	54.8 (54.4–55.2)	51.5 (51.0–52.0)	51.5 (51.0–52.0)	8.2 (7.9–8.5)	19.4 (18.8–20.0)
Prior CHD					
Yes	68.4 (65.8–71.0)	76.2 (73.3–79.1)	64.6 (61.3–67.8)	20.4 (17.7–23.1)	31.6 (27.7–35.5)
sNo	55.5 (55.1–55.8)	52.3 (51.8–52.8)	42.8 (42.3–43.3)	8.3 (8.0–8.6)	19.4 (18.8–20.0)

The value is presented in percent (95% CI). *CVD* Cardiovascular disease, *CHD* coronary heart disease

**Fig. 3:**
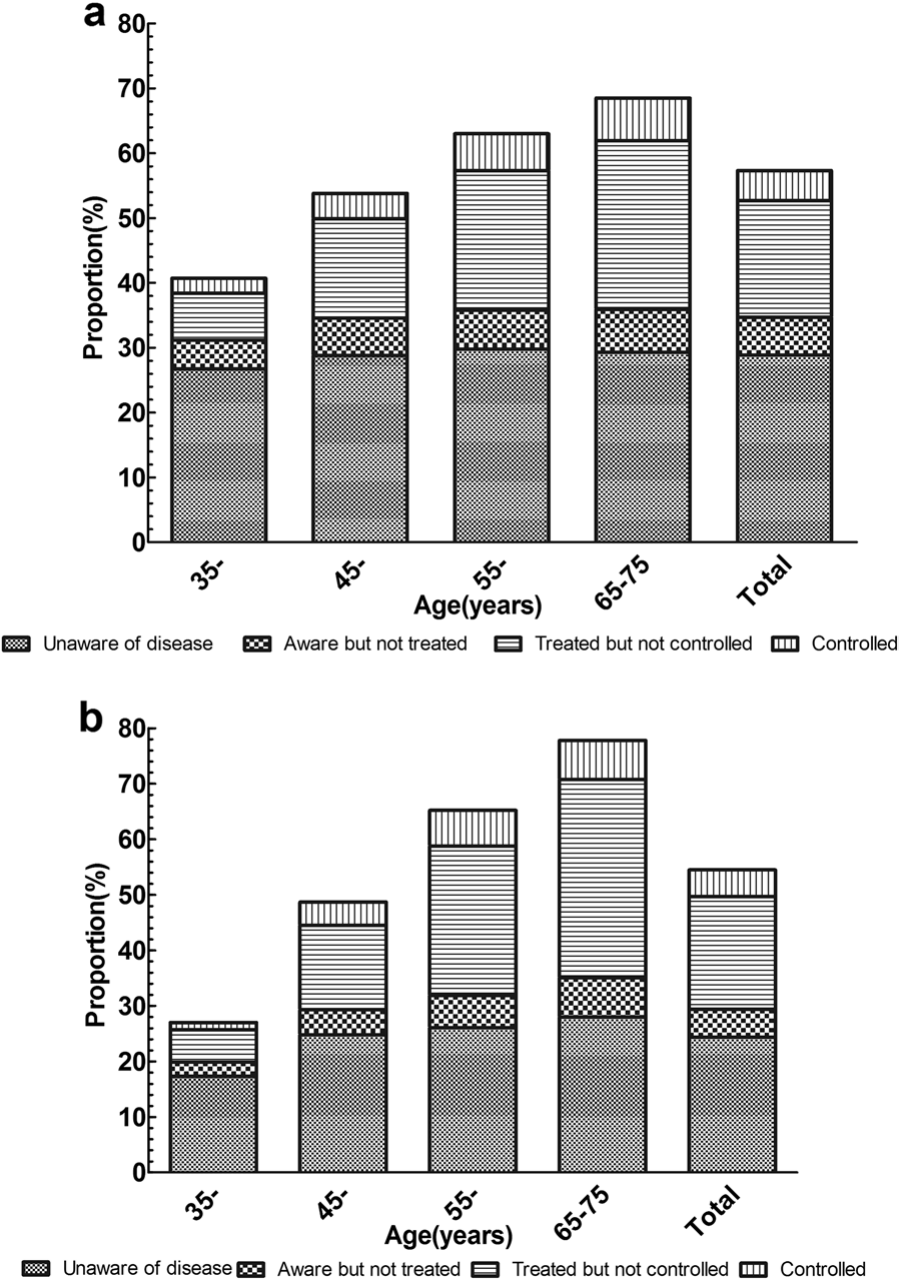
Awareness and control of hypertension among groups by sex and age. **a** Male. **b** Female

Hypertension was categorized as unaware of disease, aware but not treated, treated but not controlled, or controlled (Fig. 3). In this study population, hypertension patients accounted for 55.7%, of which 26.3% were unaware of disease, 19.3% were treated but not controlled, 5.3% were aware but not treated, and only 4.8% were controlled. The proportion of the hypertension patients and the proportion of each part increased with age. The control rate among patients with hypertension was very low among all age groups in both male and female.

### The factors associated with prevalence and control of hypertension

**Fig. 4:**
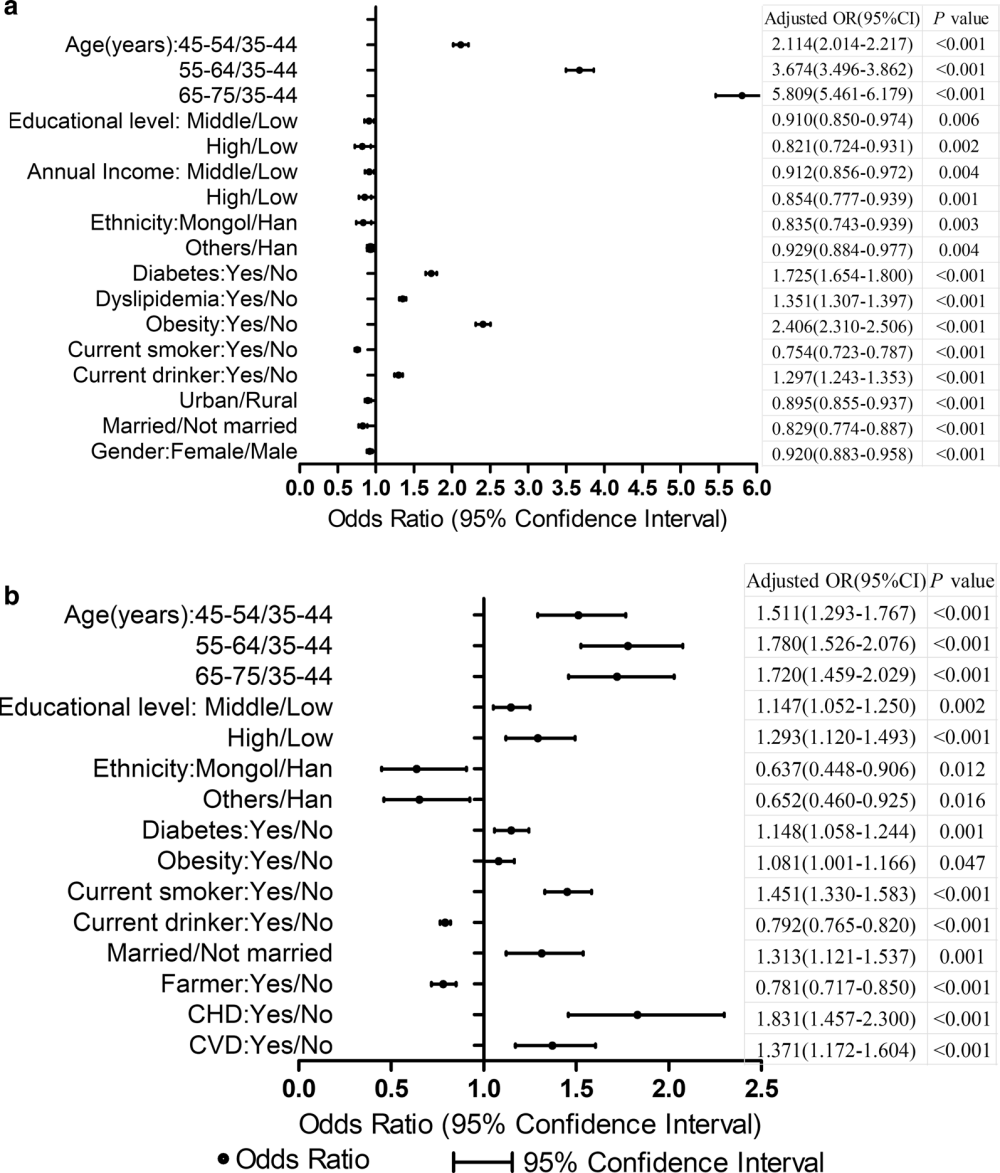
Multivariable logistic regression showing factors associated with prevalence and control of hypertension in Northern China population. **a** Prevalence. **b** Control

Using the multivariable logistic model, we identified significant determinants that were associated with the prevalence and control of hypertension (Fig. 4). Among all participants who had dyslipidemia, diabetes, obesity, Han, not married, lower educational level, lower annual income, current drinker, non-current smoker, living in rural areas, male and older were significantly more likely to be suffered from hypertension (*P* < 0.05). Patients who were farmer, Mongol, not married, current drinker, non-current smoker, younger, lower educational level, without obesity, without diabetes, without prior CHD, and without prior CVD were less likely to be controlled for hypertension (*P* < 0.05).

The odds of hypertension were 5.809 times more in participants of age group 65–75 than those in 35–44 age group (ORs = 5.809, 95%CI = 5.461–6.179, *P* < 0.001). The odds of hypertension in the Mongol were 0.835 times that of the Han (ORs = 0.835, 95%CI = 0.743–0.939, *P* = 0.003). Furthermore, hypertension was associated with dyslipidemia (ORs = 1.351, 95%CI = 1.307–1.397, *P* < 0.001); diabetes (ORs = 1.725, 95%CI = 1.654–1.800, *P* < 0.001); current drinker (ORs = 1.297, 95%CI = 1.243–1.353, *P* < 0.001), and, obesity (ORs = 2.406, 95%CI = 2.310–2.506, *P* < 0.001). Controlled for hypertension were associated with prior CHD (ORs = 1.831, 95%CI = 1.457–2.300, *P* < 0.001); older (65–75/35–44: ORs = 1.720, 95%CI = 1.459–2.029, *P* < 0.001); obesity (ORs = 1.081, 95%CI = 1.001–1.166, P = 0.047); and current drinker (ORs = 0.792, 95%CI = 0.765–0.820, *P* < 0.001).

## Discussion

To the best of our knowledge, this study presents the latest reliable information about the epidemiological situation of hypertension in Inner Mongolia, the largest province in northern China. In the first and largest population survey in Inner Mongolia, we precisely estimated the disease burden of hypertension in Inner Mongolia. In northern China, Inner Mongolia, the rates of prevalence, awareness, treatment, control, and control under treatment for hypertension was 55.7%, 52.8%, 43.3%, 8.6%, and 19.8%, respectively.

The mean SBP/DBP in our study was 140.5/84.8 mmHg (male 140.7/86.7 mmHg and female 140.4/83.4 mmHg). Compared to our results, one study conducted in Jilin Province [13], which is located in northern China and had a similar economic level to Inner Mongolia, showed lower blood pressure levels (130.5/85.0 mmHg in males, 128.3/81.7 mmHg in females). The results showed that populations had higher blood pressure in Inner Mongolia, especially SBP. There was a closely causal relationship between blood pressure and the incidence and death of cardiovascular and cerebrovascular diseases. The SBP of Asians increased by 10 mmHg, and the risk of stroke and myocardial infarction increased by 53% and 31%, respectively [18]. If we didn’t take action, the disease burden caused by hypertension would be serious, in northern China.

We found that hypertension with high prevalence and low control in northern China, which was similar to the results of other previous studies from in Inner Mongolia and northern China [11, 19–22]. The age- and sex-standardized rates of hypertension prevalence were higher than those in a 2017 report from the national report (49.4% vs. 37.2%) [6]. Although the prevalence of hypertension has already exceeded that in high-income countries, such as Australia, Canada, and South Korea, the awareness, treatment, and control rates in northern China were far inferior to those in the aforementioned countries [23, 24]. Even a study conducted in 44 low-income and middle-income countries showed that the lowest control rate of hypertension was 11.3% in Belize [25], which was higher than our study results (8.6%). In addition, most of the hypertension patients in the study were unaware, and were treated but not controlled. Therefore, it is necessary to improve the discipline level and service capabilities of the grassroots units in Inner Mongolia by standardizing the screening for hypertension and comprehensive treatment and implementing comprehensive management and control strategies. In Inner Mongolia, primary health services are in a state of higher demand for health services, but the utilization and resource allocation of primary health services are at a lower level than the whole country [26]. Moreover, the system of tiered diagnosis and treatment for hypertension has not been developed in Inner Mongolia, which could contribute to the standardized management of hypertension and enhance the efficiency of utilization of primary health resources. Therefore, the investment in the resources of the primary health service system and the utilization rate of services in the study region should be improved.

The higher prevalence of hypertension was found in our study may be explained by obesity and a high-salt diet, which were the main modifiable factors associated with hypertension. As well known, a high-salt diet is common in Inner Mongolia. A survey in Inner Mongolia showed that the salt intake was twice more than the recommended salt intake by World Health Organization (WHO) [27]. The Global BMI Mortality Collaboration reported that overweight and obesity significantly increased the risk of all-cause death in the global population [28], and were important risk factors for hypertension [4, 13, 29] The obesity rate in this study was 24.9%, while the national study in the same period reported that the obesity rate of the 35–75 years old population was 15.7% [6]. We found the obesity was associated with better hypertension awareness, treatment, and control among hypertensive patients. These findings are in agreement with He’s reports [30]. It has been suggested that obesity positively influences hypertension checking and prescription of medication for intervention, hence, higher awareness, treatment and control levels.

People who were older, male, Han, not married, living in rural areas, those with lower education, lower annual income, current drinker, non-current smoker, and coexisting conditions (diabetes, obesity, or dyslipidemia) were more likely to have a higher risk of hypertension. Among people with hypertension, those who were older, Han, not farmer, not married, current smoker, non-current drinker, those with higher education, with prior CHD, prior CVD, and coexisting conditions (diabetes or obesity) were more likely to be controlled for hypertension. Although participants of the 35–44 age group had the lowest prevalence of hypertension in our study, their prevalence was higher than the national prevalence of hypertension (32.6% vs. 22%) [6], and the 35–44 age group constituted the largest proportion of populations with high normal blood pressure. If the blood pressure of these populations was not well controlled, it will further increase the burden of hypertension. Notably, younger patients had lower awareness, treatment and control rate of hypertension, but had higher control under treatment rate of hypertension. Compared with older adult patients, younger patients lack health awareness, have fewer consultations with clinicians, have more bad habits, are less likely to adhere to prescribed medications, but they have lower blood pressure levels. If younger patients can adhere to regular medication, hypertension can be well controlled. Moreover, most guidelines advocate screening for hypertension from a relatively young age [31]. However, in our study, the participants of the 35–44 age group had the lowest hypertension awareness rate. Thus, screening strategies need to be designed to appeal to individuals of young age.

Because Mongol was the main minority ethnic group in Inner Mongolia, the ethnicity of participants was categorized into three groups including Han Chinese, Mongol, and other minority ethnic groups. Logistic regression analysis showed that the odds of hypertension in the Mongol were 0.835 times that of the Han (ORs = 0.835, 95%CI = 0.743–0.939, *P* = 0.003). However, researches by Li et al. [13, 19, 29] showed that Mongol populations have a higher prevalence of hypertension than Han populations. Different ethnic-specific genetic susceptibility, environmental exposures and the interactions between gene and environment may account for the different prevalence of hypertension [32]. A study conducted in Inner Mongolia showed that rs13306673 is a genetic factor for hypertension in the Han population but not in Mongolian population [33]. A further study between hypertension and ethnic specific genetic susceptibility is urgently needed to clarify the observation.

Current drinkers had a higher risk of hypertension prevalence, but worse control. The results of research on the relationship between smoking, drinking and hypertension are inconsistent [34–36]. Some studies showed that smoking had no clear relationship with hypertension. Many studies had shown that hypertension was related to the level of alcohol consumption [35]. However, our study demonstrated that current smoker was a protective factor for hypertension. This can be attributed to the fact that cross-sectional studies cannot determine the sequence of cause and effect and that more hypertension tends to change bad lifestyles, such as quitting smoking. We also observed that people with higher educational levels had comparatively lower prevalence and higher control rates. The phenomenon may be because that higher educational level may have a positive impact on the knowledge of hypertension prevention and control, as well as accessibility and adherence to medical treatment [37].

Several studies have found that the risk of hypertension is much higher among males than females, which was consistent with our research results [38, 39]. Compared to females, males are less concerned about the health and have more bad habits [40]. Moreover, we found a rural–urban disparity in the prevalence of hypertension. Compared with urban areas, hypertension with higher prevalence and lower awareness, treatment, and control in rural areas. This suggests that patients with hypertension in rural areas groups should be given more attention in promoting awareness, treatment and improving the management of hypertension.

## Conclusion

The results of our study suggested that hypertension in northern China is a serious problem, especially a lack of control. Even treated patients with hypertension had still not achieved adequate levels of control. The study supports broad-based opportunities to mitigate the burden of hypertension. We urgently need to develop strategies that focused on controlling the modifiable risk factors, aiming to improve the prevention and control of hypertension.

### Limitations

Because this study was a cross-sectional study, the finding cannot be used to establish a conclusive cause-and-effect relationship between risk factors and hypertension. There are some limitations. First, blood pressure value was measured in a single visit, so its value, as well as the prevalence of hypertension based partly on the measured blood pressure, may have been overestimated. Second, the study did not use representative sampling because it was not possible with such rapid, large-scale recruitment. Based on data from the total population in Inner Mongolia, we adjusted for the main asymmetrical characteristics of the study population, including age, sex, location of residence, and ethnic groups. The adjusted prevalence, awareness, treatment, and control rates of hypertension are very close to the crude rate. Third, we would expect that the rates of prevalence, awareness, treatment, and control were overestimated in our study due to the sampling bias, since the potential participants who were more concerned about their health were more inclined to respond to our study. In addition, because information about diet and exercise was unavailable in our study, the rate of treatment of hypertension could be underestimated, but the control rate would remain unaffected. Lastly, the members in a family who satisfied the inclusion criteria were selected as our study participants; as a result, the correlation of influencing factors within families may have affected the association estimation between influencing factors and hypertension.

## Data Availability

The data that support the findings of this study are available from Early Screening and Comprehensive Intervention Project for High Risk Groups of Cardiovascular Diseases of National Center for Cardiovascular Diseases, but restrictions apply to the availability of these data, which were used under license for the current study, and so are not publicly available. Data are however available from the authors upon reasonable request and with permission of National Center for Cardiovascular Diseases.

## Abbreviations

SBP: Systolic blood pressure
DBP: Diastolic blood pressure
CVD: Cardiovascular disease
CHD: Coronary heart disease
BMI: Body mass index
SD: Standard deviation
95% CI: 95% Confidence interval
